# PReS-FINAL-2300: Shrinking lung syndrome in an adolescent male with juvenile systemic lupus erythematosus

**DOI:** 10.1186/1546-0096-11-S2-P290

**Published:** 2013-12-05

**Authors:** M Rodrigues, A Teixeira, C Ferraz, I Brito

**Affiliations:** 1 Pediatrics, São João Hospital, Porto, Portugal; 2 Pediatric Nephrology Unit, São João Hospital, Porto, Portugal; 3 Pediatric Pulmonology Unit, São João Hospital, Porto, Portugal; 4 Pediatric Rheumatology Unit, São João Hospital, Porto, Portugal

## Introduction

Pulmonary involvement occurs in up to 80% of Systemic Lupus Erythematosus (SLE) patients, with decreased diffusing capacity (DLCO) and interstitial lung disease being most common.

Shrinking lung syndrome (SLS) is a rare complication, which consists in small lung volumes, elevation of the diaphragm and restrictive physiology without parenchymal or pleural involvement.

Its pathogenesis remains controversial, and anomalies such as diaphragm dysfunction, phrenic neuropathy, respiratory muscles myopathy and pleural inflammation have been hypothesized.

## Objectives

A 14-year old male adolescent was diagnosed with Evans Syndrome (autoimmune hemolytic anemia and thrombocytopenia) when he was 2 years-old, which required chronic high-dose steroids, azathioprine and splenectomy for disease control. A very low stature, osteopenia, delayed puberty and full moon facies are complications of long term steroid use.

When he was 13 years-old he presented with arterial hypertension, subnephrotic proteinuria, positive ANA and anti-dsDNA antibodies.

The diagnosis of juvenile SLE was then established, with hematological, articular (recalls swollen knees, has slight restriction of wrists), neurologic (moderate cognitive deficit and frequent headaches), and renal involvement (biopsy showed class V+II (WHO) lupus nephropathy). No evidence of peripheral neuropathy or myositis.

## Methods

For the last year, he reported increasingly worse dyspnea and pleuritic chest pain. On examination had prolonged expiration and bilateral crackles. High-resolution CT chest excluded thromboembolic complications and pleuroparenchymal changes. Echocardiogram and ECG had no signs of pulmonary arterial hypertension. Lung function tests (LFTs) showed severe restrictive pattern, small lung volumes, negative bronchodilator response and normal DLCO/Va. Despite the absence of an elevated diaphragm, these findings are highly suggestive of SLS.

## Results

Upon SLE diagnosis, treatment with prednisolone, mycophenolate mofetil, hydroxichloroquine, ACEi and support therapy was started with good results. Stable CBC, normal ESR, CRP, complement and dsDNA titres, residual proteinuria, and normal BP were attained, with prednisolone progressively reduced until 10-15 mg/day. Although chest pain resolved and lung function tests improved, he maintains marked limitation in activity due to dyspnea. Salbutamol provides symptomatic relief but LFTs seem to worsen with further steroid reduction.

## Conclusion

No treatment has been validated for SLS. Steroids are proposed as first-line therapy, along with β2 agonists. Multiple treatments have been described: steroids in association with several immunossuppressants, rituximab, theophylline and diaphragmatic pacemaker.

We propose that pulmonary findings in this case may be partially due to chronic structural changes which could explain the suboptimal response to treatment. Nocturnal non invasive ventilation was started 2 weeks ago and further treatment is being considered depending on the response.

Since the success of SLS treatment probably depends on early diagnosis and therapy, the authors emphasize that it should be suspected in any patient with autoimmune disease presenting with unexplained dyspnea.

## Disclosure of interest

None declared.

